# Current Trends in Bone Augmentation Techniques and Dental Implantology: An Editorial Overview

**DOI:** 10.3390/jcm11154348

**Published:** 2022-07-27

**Authors:** Nicola De Angelis, Stefano Benedicenti, Angelina Zekiy, Andrea Amaroli

**Affiliations:** 1 Department of Surgical and Diagnostic Sciences, University of Genoa, 16132 Genoa, Italy; benedicenti@unige.it; 2 Department of Dentistry, University Tunku Abdul Raman Sungai Buloh Malaysia, Kajang 43000, Malaysia; 3 Department of Dentistry, Trisakti University, Jakarta 11440, Indonesia; 4 Department of Orthopedic Dentistry, Faculty of Dentistry, First Moscow State Medical University (Sechenov University), 119991 Moscow, Russia; zekiy82@bk.ru (A.Z.); andrea.amaroli@unige.it (A.A.)

Dental implants and related bone augmentation problems have seen major progress since early protocols were tested in the 1980s. However, major challenges still exist concerning hard tissue augmentation procedures. Indeed, more than half of the patients show bone defects and require augmentation solutions for implant installation.

The bone augmentation procedures experience healing and maturation complexity because of the biology and physiology of soft and hard tissues, the patients’ medical risk factors, the grafting materials employed and the complexity of surgical techniques used to implant them [1].

Therefore, conventional bone grafting materials can bring disadvantages, because of the brittleness of allografts, xenografts, and alloplasts, uneasy porous forms generation, and the inability to generate precise patient-specific structures to comply with the need for precision medicine. In the meantime, autografts cannot be easily shaped to meet bony defect demand [2].

Additionally, the acceptance of bone augmentation materials can be affected by religious or life ethics—vegetarian or vegan—personal choices, as well as allergies, and refused when animal-derived products are proposed.

The challenges for researchers are, therefore, to explore novel bone graft substitutes to be used as allografts, xenografts, and alloplasts, or therapy able to prevent bone resorption and/or support bone augmentation.

Certainly, new biomaterials and their employment in the printing of 3D scaffolds are one the more interesting support structures for the future of implant dentistry.

Biodegradable natural polymers in association with bioactive and/or mechanically strong materials showed the potential to combine bioactivity and modelling. The application of 3D-printed scaffolds in tissue regeneration, and particularly in bone and sinus augmentation, and socket preservation suggested promising outcomes [3].

Behind or alongside 3D techniques, novel therapies from collaborative physical, biomolecular and pharmaceutical research could support and improve bone augmentation medical requests.

Photobiomodulation has recently demonstrated its reliability in the field of regenerative medicine. The ability of light in the visible and near-infrared spectrum to stimulate endogenous cell targets and modulate its energetic metabolism influencing the stem cell and osteo-precursor agenda was recently reviewed [4]. Promising results on cancer cells and oral microbiota have supported the PBM safeness, when adequate laser therapy parameters and delivery are employed [5,6]. Additionally, promising data were demonstrated by plasmid therapy. The p62 plasmid (also named sequestosome1/SQSTM1) was demonstrated to contrast bone loss in a mouse model of osteopenia [7].

Lastly, recent research points out the interest in dopamine behavior as an immunomodulatory mediator of several immune cells and its possible target role for drug therapy acting on osteo-inflammation and bone degeneration. Particularly, treatment with a dopamine-2-type receptor agonist is able to counteract inflammation and stimulate bone regeneration and support the transplantation of stem cell bone precursor efficiency [8].

Collectively, the recent background on bone augmentation for implant dentistry reinforces the need to develop meaningful clinical endpoints supported through the collaboration of a wide field of scientific research in biology, pharmacology, physics, chemistry and engineering. The key role of translational medicine will be mandatory in light of compliance with precision medicine and supporting the needs of clinicians, private-practitioners, and patients.
